# Rapid and Simple Buffer Exchange Using Cation-Exchange Chromatography to Improve Point-of-Care Detection of Pharmacological Agents

**DOI:** 10.3390/bios13060635

**Published:** 2023-06-08

**Authors:** Michael C. Brothers, Maegan Kornexl, Barlow Guess, Yuri Kim, Darrin Ott, Jennifer A. Martin, Dara Regn, Steve S. Kim

**Affiliations:** 1 711th Human Performance Wing, Wright Patterson Air Force Base, Dayton, OH 45433, USA; mbrothers@ues.com (M.C.B.); darrin.ott.2@us.af.mil (D.O.); 2 UES Incorporation, Dayton, OH 45432, USA; 3 Materials and Manufacturing Directorate, Wright Patterson Air Force Base, Dayton, OH 45433, USA; 4 United States Air Force School of Aerospace Medicine, Wright Patterson Air Force Base, Dayton, OH 45433, USA

**Keywords:** buffer exchange, drug detection, lateral flow assay, urinary assay, salivary assay, point-of-care assay

## Abstract

The current COVID-19 pandemic has highlighted the power, speed, and simplicity of point-of-care (POC) diagnostics. POC diagnostics are available for a wide range of targets, including both drugs of abuse as well as performance-enhancing drugs. For pharmacological monitoring, minimally invasive fluids such as urine and saliva are commonly sampled. However, false positives or negatives caused by interfering agents excreted in these matrices may confound results. For example, false positives have, in most cases, prevented the use of POC diagnostics for pharmacological agent detection; the consequence is that centralized labs are instead tasked to perform these screenings, resulting in significant delays between sampling and testing. Thus, a rapid, simple, and inexpensive methodology for sample purification is required for the POC to reach a field-deployable tool for the pharmacological human health and performance assessments. Buffer exchange is a simple, rapid approach to remove interfering agents, but has traditionally been difficult to perform on small pharmacological molecules. Therefore, in this communication, we use salbutamol, a performance-enhancing drug, as a case example to demonstrate the efficacy of ion-exchange chromatography as a technique to perform buffer exchange for charged pharmacological agents. This manuscript demonstrates the efficacy of this technique leveraging a commercial spin column to remove interfering agents found in simulant urines, such as proteins, creatinine, and urea, while retaining salbutamol. The utility and efficacy of the method was then confirmed in actual saliva samples. The eluent was then collected and run on the lateral flow assays (LFAs), improving the reported limit of detection by over 5× (new lower limit of detection of 10 ppb compared to reported 60 ppb by the manufacturer) while simultaneously removing noise due to background interfering agents.

## 1. Introduction

The advent of modern medicine has seen a significant increase in the diversity and availability of pharmacological agents. Consequentially, medication management and monitoring have become increasingly important, especially for pharmacological agents that can be abused for recreational and/or sporting purposes, such as narcotics and steroids. Cost-effective tests and diagnostics have been developed for a wide range of pharmacological agents. However, most clinical diagnostics, in an attempt to eliminate false positives and negatives, require significant sample handling and/or stringent protocols that require either trained individuals or advanced machinery. As a result, there is an increased reliance on medical diagnostic companies by many healthcare providers to execute diagnostics, as they have the infrastructure in-place, including analytical equipment (>$100,000) and trained personnel to accurately and cost-effectively execute complex biochemical assays. However, analysis of samples off-site results in a significant delay between sample collection and data reporting as samples must be transported to the central facility and must wait in a queue before being analyzed [1]. Thus, despite the assays themselves only requiring minutes to hours to complete, results typically take days to be available to healthcare providers and patients.

As a response to this, point-of-care (POC) assays for clinical diagnostics have been developed for a select number of analytes that are either highly critical for clinical care, such as blood glucose monitoring, coronavirus detection, and pregnancy detection, or are present in high concentrations in urine (protein, urea, creatinine, pH, etc.). However, issues with adulterants, false positives, and false negatives remain, thus limiting the efficacy and confidence of these diagnostics [2,3]. Therefore, to improve the access to medical information, further development of POC diagnostics is needed to expand both the number of analytes that can be measured, the accuracy of the tests, and the accessibility of these diagnostics.

Ideally, a POC diagnostic needs to (1) be able to be stored at room temperature, (2) require only a few, simple steps, and (3) have an automated analysis protocol that can be performed either by eye or by an inexpensive or ubiquitous analyzer (such as a smartphone). Assays that meet these criteria can be performed either by minimally trained individuals at local health clinics and pharmacies or by the patient themselves, providing real-time, actionable data [4]. The most common and ubiquitous POC assays rely on paper microfluidics and lateral flow principles to execute immunoassays [5]. For colorimetric/fluorometric assays, the test line either increases in intensity (classical sandwich assay) or decreases in intensity (competitive assay) as a function of analyte concentration, thus reporting on the presence/concentration of the analyte of interest. These assays are used most commonly for monitoring proteins indicative of pregnancy [6], COVID-19 [7], and flu [8], but can also be used for monitoring small molecules, such as hormones [9] and pharmacological agents [10].

While the simplicity of POC diagnostics makes them attractive, they must be able to operate in the presence of background agents, interfering agents, or masking agents present in the sample. However, in excretory fluids commonly sampled for POC diagnostics, such as urine and saliva, the concentration of these interfering agents is variable; thus, the diagnostic needs to have similar performance characteristics regardless of the composition of the excretory fluid [11]. Most relevantly, these excretory fluids vary in the concentration of interfering agents, such as protons (pH), denaturing agents (e.g., urea [12]), redox-active compounds (e.g., uric acid), and protein matrices (e.g., serum albumin and mucins). Protein-interfering agents are particularly troublesome, as they can both bind to the target and can increase the viscosity of the sample, which is known to modify flow in POC diagnostics, and thus, modify the output signal as well [13]. Thus, extensive optimization of the POC diagnostic is needed to mitigate the impact of all of these confounding interferants.

POC diagnostics for pharmacological agents are particularly challenging as many over-the-counter and pharmacological agents have similar structures. As a result, they tend to have cross-reactivity and can thus result in false positives [14]; the composition and concentration of the buffer are known to impact the selectivity of diagnostics, and are thus variables that need to be controlled [15].

Buffer selection is optimized in commercial diagnostics to improve selectivity and reproducibility [16]. However, sometimes buffer choice is limited, as some biofluids, such as saliva, require buffers with sufficient detergent/salinity to extract analytes from the surrounding matrix [17]. Dilution can mitigate buffer impacts, but it comes at a cost of sensitivity [18]. Meanwhile, the buffer exchange can mitigate the above issues, ultimately enabling one to both retain analyte and mitigate matrix effects from biological samples.

Buffer exchange is a common technique used in purification of biological macromolecules including proteins and oligonucleotides. Dialysis and size-exclusion columns are most commonly used for buffer exchange, where small molecules (below a specified molecular weight) are separated from the larger analyte either by a filtration membrane (dialysis) or by creating a tortuous path for small molecules (size-exclusion) [19]. Notably, size-exclusion columns are now available as relatively inexpensive, reusable spin-columns that require minimal equipment (e.g., PD-10 (Cytiva, Marlborough, MA, USA) and Zeba desalting columns (Thermo Scientific, Waltham MA, USA)). However, these techniques are not compatible with small molecule pharmacological agents, as many of the interfering agents, such as uric acid, urea, and salts, are similar in molecular weight to the target analytes. Therefore, an alternative method is needed to enable rapid buffer exchange for small molecule pharmacological agents.

Many pharmacological agents have positive charges at physiological pHs [20], including common drugs of abuse (e.g., fentanyl, methamphetamine, oxycodone, methadone, suboxone) and performance-enhancing drugs (e.g., ephedrine, salbutamol). Therefore, these compounds should be attracted to anionic matrices, such as sulfonates [21]; anionic matrices are commonplace in commercial ion-exchange chromatography units.

Ion-exchange chromatography is a common method leveraged for purification of proteins [22], quantitation of ions [23], water desalination [24], and other analytical separations. Ion-exchange columns are commercially available in resin form as well as in pre-packaged columns. Notably, ion-exchange chromatography should enable retention of positively charged pharmacological agents and removal of neutral (e.g., urea) and negatively charged (e.g., creatinine, uric acid, ascorbic acid [25]) interfering agents as well as most common interfering proteins, such as serum albumins and mucins (isoelectric point <7). Additionally, the target analyte could then be eluted into a known buffer composition that can be used directly in the assay. This proposed method for performing buffer exchange and sample isolation, however, has not been demonstrated to-date for drugs of interest in simulant biologically relevant matrices.

Salbutamol (albuterol) presents a useful test-case, as while many immunoassays for the analyte exist, the only lateral flow assay (LFA) commercially available to researchers and clinicians for detection of albuterol is designated for research use only. Salbutamol is a positively charged β2 agonist that has use in humans and in livestock. Most commonly, it is used as a bronchodilator for humans to treat asthma, but also as a performance-enhancing drug in endurance sports. Salbutamol is excreted from the body in urine both as an unadulterated drug and as select metabolites. Salbutamol is currently monitored for in performance athletes [26] primarily using liquid-chromatography/mass-spectrometry (LC-MS), the current gold standard for identifying compounds and determining concentrations of performance-enhancing drugs [27]. Interestingly enough, salbutamol is also commonly used in livestock to promote or improve feed efficiency and lean muscle mass growth [28]. Therefore, an improved rapid method to detect salbutamol in urine, saliva, and other matrices would be valuable for anti-doping controls, medication monitoring, and food contamination monitoring.

In this communication, we explore the efficacy of a simple cation-exchange column to enable rapid sample purification/preparation in both simulant urine as well as human saliva. First, we demonstrated the impact of specific interfering agents from simulant urine [29] on LFA outputs. Second, we optimized a protocol to perform buffer exchange leveraging commercially available cation-exchange columns, including demonstrating that the analyte of interest is retained and that interfering agents are removed. Third, we determined the LFA output as a function of analyte concentration to obtain a concentration-dependent calibration curve and to determine the new lower limit of detection (LLOD) of the LFA leveraging the method described in this manuscript; the new LLOD of 10 ppb is over 5× lower than the reported 60 ppb by the manufacturer. Therefore, the method described in this manuscript provides a new, low-cost, simple method to remove interfering agents from samples and to better control sample buffer conditions for POC diagnostics.

## 2. Materials and Methods

### 2.1. Sourcing of Chemicals

Unless otherwise specified, all chemicals used in this study were purchased from Sigma Aldrich and were used without further purification. De-identified human saliva was used from previous studies as a test matrix.

### 2.2. Preparation of Simulant Urines (SUs) and Components

Experiments were performed using four urine simulants to test the impact of differing interfering factors (Table S1). Unless specified, all urine simulants were pH-buffered to pH 7.4. Concentrated urine simulant (CUS) contained the maximum concentration of all interfering agents typically found in urine (Table S1). Salt-only urine simulant (SOU) contained only the maximum concentration of salinity without any additional adulterants. Finally, dilute urine was simulated with ultrapure water to create a water urine simulant (WUS) to ensure assay function in case samples were provided from an over-hydrated individual or were tampered with by diluting in water.

### 2.3. Preparation of Albuterol Stocks

All albuterol stock solutions (10,000 ppm) were prepared by dissolving 10 mg of albuterol sulfate into 1 mL of either CUS, SOU, or WUS. The stock was then diluted 100:1 to make a 100 ppm and a 1 ppm stock solution of albuterol in the desired buffer/urine simulant. Lower concentrations used in the assay (80 ppb, 40 ppb, 20 ppb, 10 ppb, 5 ppb, 2.5 ppb, 1.25 ppb, 0.625 ppb, 0.3125 ppb) were then made by serial dilution and used directly in the study without further modification.

### 2.4. Ion-Exchange Chromatography for Removing Sample Interferants

Pierce™ cation-exchange columns (mini) were purchased from Thermo Scientific and were used without further modification. The ion-exchange chromatography protocol to remove sample interferants was performed as follows: First, 400 µL of sample was added to the top of the column. The column was centrifuged and the eluent was removed. Next, 400 µL of 2 mM phosphate buffer at pH 7.4 (no saline) was added to the column and centrifuged. The eluent was removed, and the process was repeated twice more to remove any residual contaminants. The columns were then loaded with 50 microliters of HEPES Buffered Saline (HBS) consisting of 50 mM HEPES, 1 M NaCl, pH 7.4 to elute the albuterol from the column. The eluent was collected, diluted by 4× in water (final salinity of 250 mM NaCl) and then run on the LFA without further modification.

### 2.5. Lateral Flow Assay for Albuterol

LFAs to detect albuterol (salbutamol) were acquired from ELabScience (Houston, TX, USA). The assays, outside of the pre-centrifugation step for urine, were performed as described in the manual. In short, 50 µL of sample was added to the LFA. The assay was allowed to proceed for 10 min before being visually checked for quality control purposes. The LFA strips were then allowed to dry overnight to remove any trace water from the LFA. The strips were then placed into an ESEQuant LF3 LFA reader and read using the LFStudio data acquisition software. The reflectance values of the LFA were then obtained using an excitation wavelength of 365 nm and an emission wavelength of 500 nm. Scans (x-pos 44–56 mm) were taken at two y-values (y-pos 3 and 4.5 mm) to improve data quality. Data were exported from LFStudio as a multi-sheet Microsoft Excel document, analyzed using the Python programming language (Appendix A), and verified with manual analysis.

### 2.6. Data Analysis and Drift Correction

Data analysis was performed using a custom python script. In short, a linear baseline drift correction was applied to account for the dye-front and to improve the reproducibility of the results. More details on the analysis and the program can be found in the Supporting Information and in Appendix A.

### 2.7. Analysis of Test Line and Noise

To determine the absolute intensity of the readings from the LFA strips, a custom peak analysis protocol (Appendix A) was applied to all drift-corrected intensity data taken from the ESEQuant LR3 reader from Qiagen (Germantown, MD, USA) reader. This protocol first calculated the raw absorbance values for the test line, calculated as the minima observed between 47 and 49 mm and the “noise”, calculated as the minima between 49.4 and 52.4 mm. The absolute heights of both the test line and the noise were measured by subtracting the reflectance value from the baseline (55.5–56 mm).

### 2.8. Calculation of Limits of Detection Using T-Test

The outputs of the test line were compared to the blank using a two-tailed *t*-test in Microsoft Excel. In short, each set of test line outputs for a given concentration was compared to the outputs for the blank for a given urine simulant or for the aggregate across all urine simulants. The *p*-value was recorded for each of these analyses. The result was considered statistically significant if *p* < 0.001.

### 2.9. Testing for Small Molecule Interfering Agents in Samples

The presence or absence of select interfering agents was tested using commercial assays. Commercial test strips were used to test for urea (QuantiQuik Urea Quick Test Strips, BioAssay Systems, Hayward, CA, USA) and creatinine (VeriCheck U-7 SVT, Vericheck, Barrie, ON, Canada) in pre- and post-processing of samples to optically test whether the interferents were present or absent.

Each test strip had 20 µL of sample added directly onto the sample pad, resulting in complete wetting of the sample pad. The color pre- and post-wetting of the sample was recorded via use of digital photography. No further analysis (quantitation) was performed.

### 2.10. Testing for Proteins in Samples

The presence or absence of proteins in samples or filtrates was determined using a bicinchoninic assay (BCA) (BCA Protein Assay Kit, Sigma Aldrich, St Louis, MO, USA). Samples and fractions (in triplicate) were used in the assay without further dilution. A standard curve of BSA was generated using 2-fold serial dilutions (2 mg/mL to 25 µg/mL) and added to a clear, 96-well plate alongside the unknown samples. The BCA analysis working reagent was then created (50 parts A to 1 part B) immediately before use and added to the wells (200 µL per well). The samples were then mixed for 20 min and the absorption values at 564 nm were acquired. The absorbance values in the sample wells were then directly compared to the standard curve.

## 3. Results and Discussion

### 3.1. Spin-Column Isolation of Pharmacological Agents

To eliminate false positives and matrix effects, a rapid, simple method is needed to isolate pharmacological agents from the surrounding biological matrix. Since many pharmacological agents have a net positive charge, ion-exchange chromatography has traditionally been leveraged to bind and separate pharmacological agents [30]. Alternatively, ion-exchange chromatography should allow for a rapid, inexpensive method to remove interfering agents and control buffer composition. However, a method has yet to be developed or demonstrated that rapidly isolates pharmacological compounds from the surrounding matrix leveraging ion-exchange chromatography.

**Figure 1:**
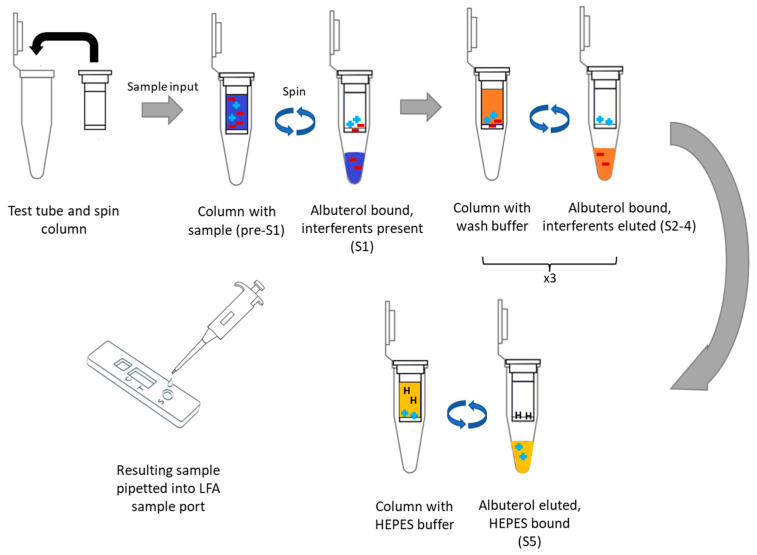
Illustration of spin column method for simple buffer exchange of pharmacological agents leveraging commercially available cation-exchange columns.

The following process was developed and used in this manuscript as illustrated below to separate interfering agents in biological samples from the pharmacological agent albuterol (Figure 1). First, the biofluid containing the positively charged pharmacological agent is loaded onto the column, immobilizing the pharmacological agent electrostatically. Then, the column is washed with 2 mM phosphate buffer, pH 7.4 to remove loosely bound interfering agents (such as proteins and lipids). Upon removal of all interfering agents, the pharmacological agent is then released through a series of washes with a high salinity buffer; the sodium ions outcompete the pharmacological agent for the negatively charged sites. The eluent is then collected, diluted four-fold to prevent salinity interferences with the assay, and then added to the LFA for quantitation. The assay output from each step is visualized in the Supporting Information (Figure S1). As can be observed in the eluent, not all of the agent is captured on the column. However, once the residual sample is cleared, the column retains the analyte when rinsed with phosphate buffer (eluents 2–4). The captured pharmacological agent is then released by the addition of one aliquot of buffered 1 M NaCl. Salinity is known to be an interferent in immunoassays, including LFAs [31]. Therefore, the eluent was diluted into 150 µL of water to reduce the salinity to 250 mM (~2× physiological) before being analyzed by the LFA.

### 3.2. Identification of Key Interfering Agents for Albuterol LFA

Buffer composition has a significant impact on the output of immunoassays and LFAs [31]. However, not all components of the sample matrix actually interfere with the assay. Therefore, a study was performed to determine which interfering agents (including pH) most impacted the output of the albuterol LFA. Buffers were made where both the pH was modified and/or urine contaminants were added. LFAs were then run, and the outputs were recorded and analyzed. As expected, urea and salt, both known antibody/protein denaturants, had the greatest impact on the LFA output (Figures S2 and S3). Even sub-physiological concentrations of urea impacted the test line value in the absence of albuterol (Figure S3); at higher concentrations, a near-linear decrease in the test line reflectivity was observed as a concentration of urea, providing a significant and likely source of false positives if the assay had urea present. No other agents substantially changed the reflectivity of the test line. Interestingly, while no visible streaking or speckling was observed when just urea was added to the sample, the urine simulant, which contained additional salts and contaminants, did cause the LFAs to have visible streaks, speckling, and discoloration. While in theory, speckling of the test strip could be mitigated, it still would likely make analysis, especially automated analysis, more complicated. pH buffering can occur through the addition of buffers in powder form (such as bicarbonate tablets). However, the removal of urea is non-trivial and remains to be addressed. Urea, a small molecule (60 Da), cannot be removed from albuterol (239 Da) based on size. Fortunately, urea has no net charge, while albuterol has a +1 charge, thus making ion chromatography a likely solution to separate urea from albuterol.

### 3.3. Validation of Sample Clean-Up Using Ion-Exchange Chromatography

**Figure 2:**
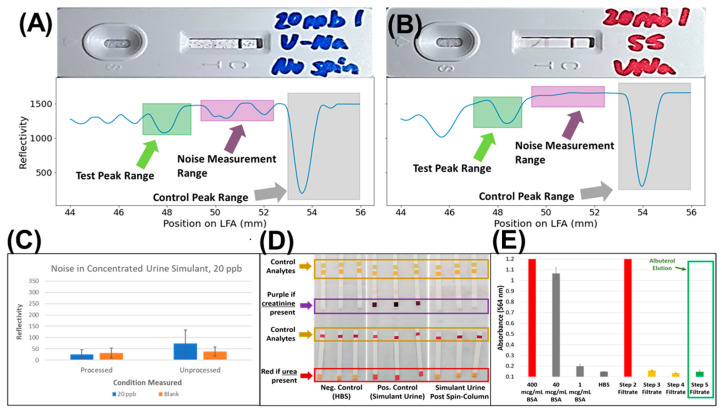
Removal of select interfering agents from pharmacological agents using cation-exchange chromatography. LFAs and corresponding optical read-outs from concentrated urine simulant pre (**A**) and post-processing (**B**) demonstrate a reduction in the noise (**C**). Cation-exchange chromatography removes select interfering agents, including creatinine and urea (**D**) and BSA protein (**E**).

In order to test the worst-case scenario, where the LFA must perform in the presence of relatively high concentrations of interfering agents, a highly concentrated simulant urine (CUS) was created (Table S1). The CUS sample, spiked with 20 ppb of albuterol, was then run on the albuterol LFA. Most notably, while the test line did disappear, indicating the competition assay was still functional, the test strip turned blue and demonstrated substantial artefacts (Figure 2A). The effect of the interfering agents was also observed on the LFA reader, as demonstrated by the observed noise peaks, which in some instances can be of the same magnitude if not larger than the test line itself (Figure 2A). In comparison, the processed samples demonstrated no change in color and/or visible speckling (Figure 2B). The ESEQuant reader confirmed this, as no discernable noise peaks were present (Figure 2B), and the only additional peak observed corresponded to the dye-front in the assay. Fortunately, this peak could be identified and mitigated by acquiring the read-out at two distinct *y*-axis values to determine if the peak position was at two different positions (dye-front) or was at the same position (test line).

In theory, the region between the test line and the control line should be flat, with any peaks attributed to noise caused by contaminants or a byproduct of the assay. Analysis of the region between the two peaks demonstrated that the processed samples exhibit far less noise than their unprocessed counterparts as measured by the intensity of noise peaks in the region between the test and control peak (Figure 2C), even when no drift correction is applied (Figure 1 and Figure S4). This indicates that removing denaturants and other impurities improves the confidence in the final LFA readout, which implies that usage of the described filtration protocol can improve the confidence in results.

Biofluids contain an array of interfering agents, and thus, it is necessary to define which classes of interfering agents are removed during the sample clean-up method described in this manuscript. Among the more abundant interferents in urine are creatinine (negative charge), urea (no charge, denaturant), and serum albumin (globular protein). These compounds also serve as overall representative compounds to demonstrate the efficacy of removing interfering agents that do not have a net positive charge. Therefore, a urine simulant containing an elevated concentration of urea (10 mM), creatinine (120 µM), and bovine serum albumin (400 mg/mL BSA) was run through the spin-column protocol. Commercially available urinary test strips were used to monitor the removal of urea and creatinine from the samples using the protocol described in this manuscript. As demonstrated, baseline HBS buffer produced no color change. Our simulant urine spiked with high concentrations of creatinine and urea produced strong colorimetric responses. As expected, that same simulant urine run through the buffer-exchange protocol caused both test strips to report creatinine and urea concentrations at baseline levels (Figure 2D). Similarly, a BCA assay was performed pre- and post-buffer exchange to monitor for the presence of protein (Figure 2E). The results (Figure 2E) demonstrated that nearly all of the protein was removed by the second phosphate buffer rinse step. By the elution step, it was at or near baseline. Notably, this test demonstrated that serum albumin, the most abundant protein in blood, saliva, and urine, could be virtually eliminated from biological fluids; this result also bodes well for removing the negatively charged mucins that are in high abundance in saliva [32].

### 3.4. Quantitation of Salbutamol Using a Commercially Available LFA

While in many cases, identifying the presence or absence of a pharmacological compound is sufficient, quantitation is still useful to both establish that the pharmacological agent is above a minimum threshold concentration to avoid false positives; law enforcement and doping controls most commonly have strict limits to not punish inadvertent exposure. Additionally, quantitation of a pharmacological agent in urine or saliva correlates back to the blood concentration, albeit with a variable dilution factor. Therefore, we explored whether the LFA could enable quantitative outputs.

**Figure 3:**
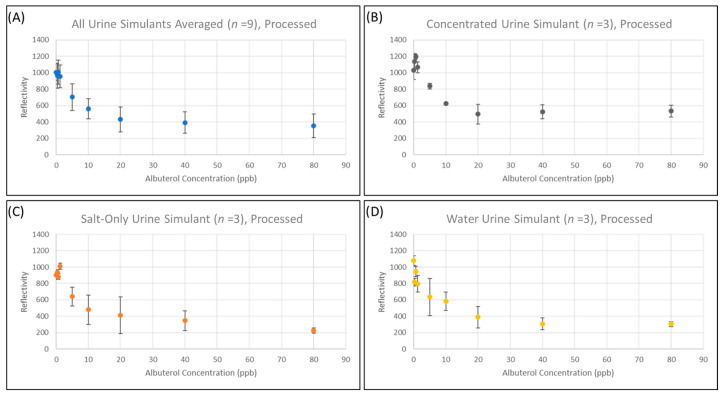
Comparison of LFA outputs versus albuterol concentration (ppb) for selected urine simulants. Aggregated LFA readout values from all urine simulants (*n* = 9) (**A**), from concentrated urine simulant (**B**), high salt only urine simulant, (**C**) and dilute urine simulant (**D**).

To determine if our method enabled a dose-dependent response, we created albuterol standards (80 ppb to 0.3 ppb) in three different urine simulants; these three different urine simulants represented diluted urine (water), concentrated urine (high salinity), and concentrated urine (high concentrations of all interferants, Table S1) that contained varying degrees of salinity and interfering agents. The standards were then processed as described above and analyzed. The goal of this test was to determine if the same outputs and standard would be obtained regardless of urine composition when leveraging the method described in Figure 1 and the analysis described in the methods. As hypothesized, the response curves between concentration and absorbance were similar regardless of the urine simulant tested after processing using the method described in this manuscript (Figure 3). In all cases, saturation of the LFA appeared to occur for concentrations of albuterol greater than 20 ppb; concentrations below 20 ppb have a signal response change as a function of concentration. As can be observed, quantitative curves can be read with reasonably low error values for a LFA (>18%) at concentrations above 20 ppb (near the saturation point of the assay) regardless of condition.

A *t*-test was then used to determine the LLOD of the assay. A *p*-value threshold of 0.001 was used as the LLOD for the *t*-test. The results of the *t*-test are reported in the Supporting Information (Table S2). Notably, according to this analysis, the LLOD reported is 10 ppb regardless of urine simulant composition, supporting that cation-exchange chromatography can mitigate matrix effects.

Another key limiting factor in deploying LFAs in the clinical setting is the assay-to-assay reproducibility, especially when adding additional steps. The standard deviation of the test line absorbances was acceptable (SD < 100 Reflectance Units ((RU)) both when the LFA outputs from all starting urine simulants tested were aggregated (Figure 3A, *n* = 9) and when the urine simulants were plotted separately (Figure 3B–D, *n* = 3). This further supports that cation-exchange chromatography is useful for sample clean-up regardless of urine composition. Additionally, the error in the readout does not appear to change as a function of analyte concentration, indicating that test strip variability likely accounts for the observed error regardless of urine simulant or albuterol concentration.

From a pragmatic, real-world standpoint, albuterol is typically observed for chronic inhaler users in the 100’s of ppb [33]. For individuals who are only taking a single dose [34], the concentrations are likely to not even be apparent by the most sensitive methods (LC-MS). Additionally, the cutoff threshold for drug testing even for pharmacological agents is typically 1 ppb [35]. Therefore, a LLOD of 10 ppb is more than sufficient, especially for the ease of use and the portability of the methodology, as it is by orders of magnitude less than what would typically be seen in human urine for individuals that are using inhalers on a regular basis.

### 3.5. Proof-of-Concept Demonstration in Saliva

**Figure 4:**
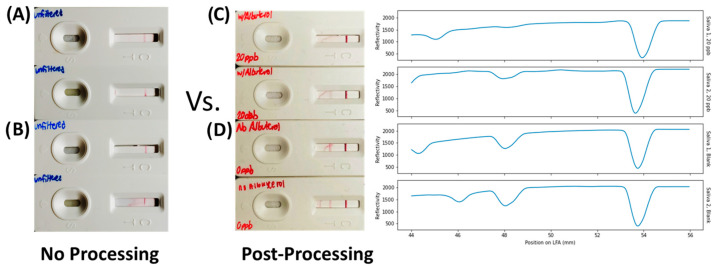
Comparison of LFA readout from two human saliva samples. LFAs with no processing return the same positive result if the samples contain 20 ppb albuterol (**A**) and 0 ppb albuterol (**B**). In comparison, processing using the method in this paper results in a clear signal when 20 ppb of albuterol is added to the sample (**C**) compared to when no albuterol is added (**D**) using either visual inspection (**left**) or an optical reader (**right**).

Saliva, due to its high protein concentration and thus viscosity compared to urine, provides a complementary challenging matrix for analyte detection. Therefore, two previously collected, de-identified saliva samples were removed from storage and split. For each saliva sample, one aliquot had 20 ppb of albuterol added to the sample while the other remained unadulterated. The samples were then processed using cation-exchange chromatography followed by LFA analysis. The main difference between the saliva and simulant urine samples was that saliva had a significantly increased viscosity; the consequence is that a higher relative centrifugal field (RCF) (>10,000) was needed to process the more viscous sample through the cation-exchange column (first addition and centrifugation step). Other than increasing the RCF for the initial centrifugation step, no additional changes to the protocol were needed. As can be clearly seen, both saliva samples demonstrated a false positive response to albuterol (Figure 4) without processing. However, upon processing using our method, 20 ppb of albuterol could be clearly detected both by the naked eye as well as using the LFA reader (Figure 4). Interestingly, the test peak for the blank signal did decrease in both samples, thus implying that a separate calibration curve is likely required for each chosen matrix. The authors hypothesize that some residual cross-reactivity with interfering agents, such as positively charged small molecules with similar sub-structures (i.e., choline), may explain the observed reduction in the test peak in the absence of albuterol compared to simulant urines.

## 4. Conclusions

Here, we demonstrate a simple, rapid, and robust method to isolate positively charged pharmacological agents from both simulant and actual biofluid samples leveraging a commercially available cation-exchange matrix. Through this effort, we were able to demonstrate an improvement on the reported LLOD by at least a factor of five in simulant urine while simultaneously improving the confidence in the result and enabling automation of data analysis. We prove that the process described in this manuscript also works for detecting pharmacological agents in saliva samples, providing a simple, efficient way to isolate charged small molecules from protein-rich matrices. Future efforts can automate and simplify the current manual process either by incorporating directly into a staged LFA, by modifying the column to operate using pressure instead of centrifugation (either positive or negative), or by leveraging paper-based ion-exchange matrices [36]. In conclusion, the simplicity of the method described in this manuscript will enable more POC assays to operate in urine, saliva, and other biofluids at a higher degree of confidence, enabling more assays to be performed at the site of care; the end result should be a decrease in the time and cost to analyze samples, resulting in improved health outcomes.

## Data Availability

Not applicable.

## Supplementary Materials

The following supporting information can be downloaded at: https://www.mdpi.com/article/10.3390/bios13060635/s1, Methods: Automated Data Analysis and Drift Correction, Figure S1: Albuterol Elution Throughout the Filtration Process, 80 ppb Albuterol; Figure S2: Impact of Interfering Agents on LFA Test-Line Peak Height with 0 ppb Albuterol (Max Concentration Observed in Urine, Table S1); Figure S3: Impact of urea concentration of test line peak height (*n* = 3) Figure S4: Noise and Error Removal via Baseline Correction, 20 ppb Albuterol. Table S1: Urine Simulant Compositions and Formulas; Table S2: Two-Tailed *t*-Test Results, Varying Albuterol Concentrations.

## Appendix A

Custom software developed and used for analysis of the LFA strips is found at the following website: https://github.com/knutmeg/LFA_Analysis.git.
