# Supplementary material for: Rapid and Simple Buffer Exchange Using Cation-Exchange Chromatography to Improve Point-of-Care Detection of Pharmacological Agents
Source: Biosensors (Basel). 2023 Jun 8;13(6):635. doi: 10.3390/bios13060635 (PMC10296204; doi:10.3390/bios13060635)
Supplement: Supplementary file 1 [file biosensors-13-00635-s001.zip › biosensors-2409784-supplementary.pdf]

biosensors
MDPI

Supporting Information

Rapid and Simple Buffer Exchange Using Cation-Exchange Chromatography to Improve Point-Of-Care Detection of Pharmacological Agents

Michael C. Brothers [1,2,†], Maegan Kornexl [1,2,†], Barlow Guess [1], Yuri Kim [1,2], Darrin Ott [1], Jennifer A. Martin [3], Dara Regn [4] and Steve S. Kim [1,*]

Methods: Automated Data Analysis and Drift Correction

A residual dye-front was observed in the assays that impacted the background absorbance of the LFA. Therefore, a background-correction protocol was applied to eliminate artefacts from the dye-front. The correction described below was conditionally applied to datasets using a custom python program (**Appendix A**).

First, the minimum reflectance between 48.9 and 49.9 mm of the LFA reading, corresponding to a region between the control and test lines and the baseline, measured, as the average, between 55.5 and 56 mm. If the difference was less than 50 absorbance units, the data file was output without further modification (no drift correction). If the difference was greater than 50 absorbance units, then background correction was applied starting at the maximum reflectance (corresponding to the beginning of the dye-front) between 49.9 and 50.9 mm position.

A linear correction was used to correct for the background. The slope of the background correction was calculated as follows: the position and reflectence for baseline values for the region adjacent (maximum reflectence 1–2 mm downstream) to the test (P_T, I_T) and the control peaks (P_C, I_C) on the LFA was identified and recorded. The background correction factor (BCF) was then calculated using the formula

$$BCF = \frac{I_c - I_T}{P_c - P_T}$$

The program then calculated both the optimal position to begin linear background correction based on the location of the start of the dye-front, determined by a linear deviation of the background from baseline.

The final corrected values were calculated using the formula

$$I_n + DCF * PCF_n$$

where *n* represents a defined measurement unit (which in practical terms, is the difference between positional measurements in the Excel sheet) and its corresponding values.

After analysis, the program automatically output an Excel sheet to the working directory containing the new, background-corrected data for each LFA input in a table-based format.

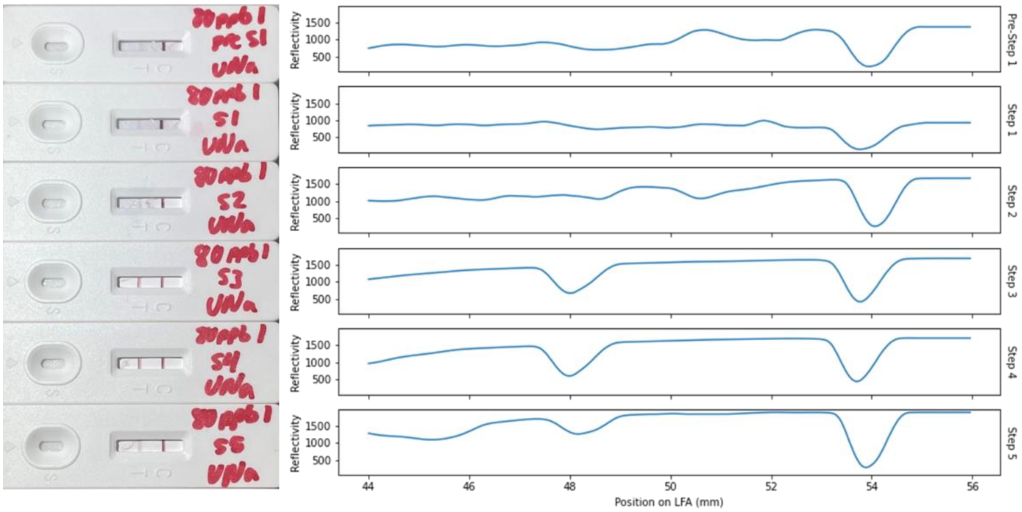

Figure S1. *Albuterol Elution Throughout the Filtration Process, 80 ppb Albuterol.*

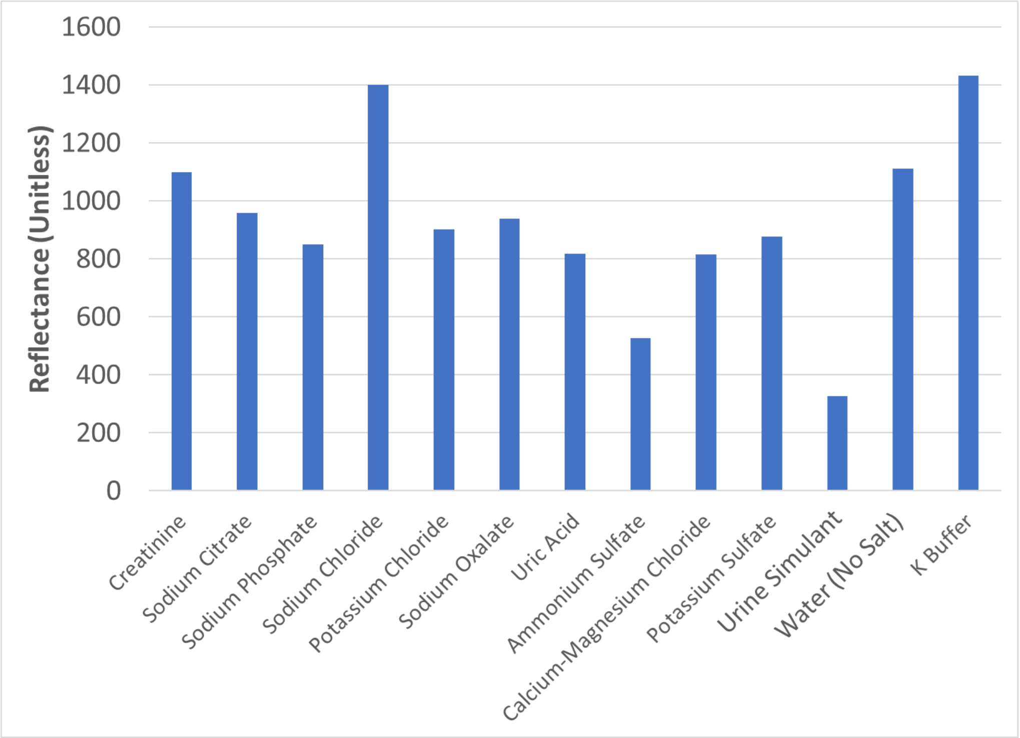

Figure S2. *Impact of interfering agent on peak height (max. concentration observed in urine,* ***Table S1).***

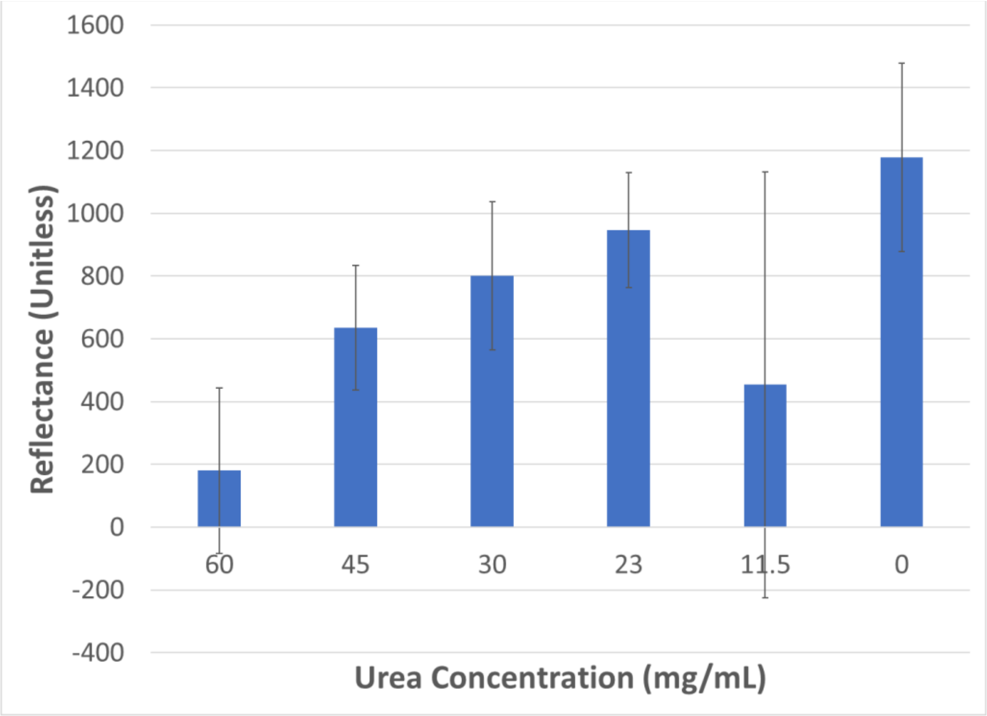

Figure S3. *Impact of urea concentration of test line peak height (n=3)*

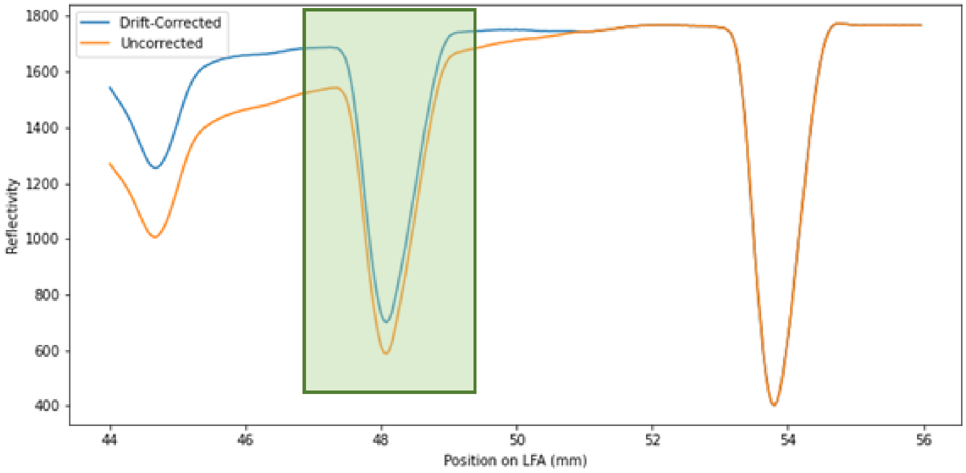

Figure S4. *Noise and error removal via baseline correction, 20 ppb albuterol.*

Table S1. *Urine simulant compositions and formulas.*

	Ultrapure Water Simulant	Salt-Only Urine Simulant	Concentrated Urine Simulant	Minimum	Maximum
**pH (unit)**	7.4	7.4	7.4	4.5	8
**Urea**	0	0	750	80	750
**Uric Acid**	0 mM	0 mM	5.95 mM	N/A	4.46 mmol/day
**Creatinine**	0 mM	0 mM	32.7 mM	5.31 mmol/day	25.95 mmol/day
**Citrate**	0 mM	0 mM	7.81 mM	1.15 mmol/day	6.20 mmol/day
**Sodium**	3 mM	300 mM	300 mM	41 mmol/day	227 mmol/day
**Potassium**	0 mM	10 mM	100 mM	17 mmol/day	77 mmol/day
**Ammonium**	0 mM	0 mM	70 mM	15 mmol/day	56 mmol/day
**Calcium**	0 mM	1 mM	1 mM	N/A	6.24 mmol/day
**Magnesium**	0 mM	1 mM	1 mM	2.10 mmol/day	11.07 mmol/day
**Oxalate**	0 mM	0 mM	0.6 mM	0.05 mM	0.6 mM
**Sulphate**	0 mM	60 mM	60 mM	3.5 mM	60 mM
**Phosphate**	2 mM	2 mM	60 mM	10 mM	60 mM
*Balance of Ions are Chloride Ions.					

Table S2. *Two-tailed t-test results for LFA outputs for urine simulants (top row) at concentrations of albuterol (left column).*

	All Simulants (N=9)	CUS (n= 3)	SOU (n= 3)	WSU (n= 3)
**80 ppb**	1.41397E-08	0.007720478	2.97023E-05	4.64373E-05
**40 ppb**	1.25518E-08	0.003508786	0.004700637	0.001143253
**20 ppb**	2.13736E-07	0.004854305	0.066178323	0.02368716
**10 ppb**	3.92623E-07	0.025025547	0.058480879	0.019529799
**5 ppb**	0.000346207	0.105153485	0.034637666	0.178559389
**2.5 ppb**	0.263500569	0.825485512	0.643840726	0.271035109
**1.25 ppb**	0.424748824	0.684248017	0.034124854	0.194744679
**0.625 ppb**	0.956305466	0.139926562	0.590593266	0.401810089
**0.3125 ppb**	0.513233281	0.275435638	0.368710052	0.076955912
